# A self-organising biomimetic collagen/nano-hydroxyapatite-glycosaminoglycan scaffold for spinal fusion

**DOI:** 10.1007/s10853-017-1229-9

**Published:** 2017-07-28

**Authors:** Aman Sharma, David Brand, Jeremy Fairbank, Hua Ye, Chris Lavy, Jan Czernuszka

**Affiliations:** 1 Department of Materials Science, University of Oxford, Parks Road, Oxford OX1 3PH, UK; 2 Oxford Spinal Unit, Oxford University Hospitals NHS Trust, Nuffield Orthopaedic Centre, Windmill Road, Oxford OX3 7HE, UK; 3 Oxford Institute of Biomedical Engineering, Old Road Campus Research Building, University of Oxford, Oxford OX3 7DQ, UK; 4 Connective Tissue Research Group, Collagen Core, Department of Medicine, Veterans Affairs Medical Center, Memphis, TN 38163, USA

## Abstract

The use of spinal fusion surgery as a treatment for degenerative spinal conditions and chronic back pain is increasing. However, this technique requires use of a bone grafting material to fuse the vertebrae, traditionally autologous bone, which consists of an optimal combination of osteogenic cell precursors, extracellular matrix proteins and mineral components. To date, this remains the ‘gold standard’ material but its supply is limited and is associated with a number of clinical and ethical difficulties; consequently, various combinations of cells with biological scaffold materials have been tested but have failed to achieve fusion rates even comparable to autologous bone. We successfully fabricated a novel collagen-based scaffold using self-organising atelocollagen combined with nano-hydroxyapatite and chondroitin sulphate, cross-linked by microbial transglutaminase. The scaffold was characterised using a range of imaging, chemical composition and thermal analysis techniques. It was found to exhibit appropriate stiffness and suitable pore size for the adhesion, growth and differentiation of MSCs. The low toxicity makes it suitable for clinical application, and its slow degradation profile would enable the scaffold to promote bone growth over an extended period. This material therefore shows promise for clinical use in spinal fusion and other procedures requiring the use of bone grafts.

## Introduction

Spinal fusion surgery is increasingly performed for the treatment of chronic back pain and other degenerative spinal conditions [1]. A major determinant of successful fusion is the bone graft. The current ‘gold standard’ graft material in spinal fusion surgery is autologous bone (commonly iliac crest bone) which contains critical components: a bony scaffold; growth factors in the bone extracellular matrix (ECM); and osteoprogenitor cells within the bone marrow. However, the harvesting of such graft material is associated with significant complications and the graft itself is of limited availability, which has led to the development of alternative graft materials. Existing synthetic grafts have failed to achieve fusion rates superior to or even comparable to autologous bone, and continuing efforts are needed to identify better grafts.

Native bone is largely made up of a soft organic matrix incorporating a highly substituted crystalline hydroxyapatite. The organic component consists largely of collagen, which is the most abundant protein in the ECM of the human body, and consequently has been studied extensively as a scaffold material in orthopaedic surgery. Collagen is non-toxic, biodegradable and has excellent osteoconductive properties. However, it lacks any osteogenic or osteoinductive properties. Its biodegradability depends on its resistance to collagenase, which is in turn dependent on the amount of intermolecular cross-linking, which increases the biodegradation time of collagen [2]. The biological half-life of collagen scaffolds can therefore be manipulated to suit different applications.

Although over 90% of the organic matrix of bone is composed of type I collagen, the remainder is made up of a heterogeneous mixture of over 200 other proteins, one of which is chondroitin sulphate, which occupies the spaces within newly developing bone and has been shown to act as an osteogenic stimulant. It could therefore have a positive role in promoting bone formation within a scaffold.

Osteoprogenitor cells within the bone marrow are a critical component of bone grafts, and the combination of human mesenchymal stem cells (MSC) with an osteoconductive scaffold and/or osteoinductive factors has been shown to increase their osteoblastic differentiation and hence promote bone formation. To date, two studies which used bone-marrow-derived MSCs in spinal fusion surgery have shown encouraging results when compared to iliac crest bone grafts, supporting the potential use of MSCs in spinal fusion surgery [3, 4].

We hypothesised that the osteogenic capability of MSCs could be optimised by their combination with collagen, hydroxyapatite and chondroitin sulphate, and such a scaffold could be developed into a graft for use in spinal fusion surgery.

## Materials and methods

### Materials

Bovine atelocollagen Type I (gift from Dr D. Brand, Veterans Affairs Medical Center, Memphis, TN, USA) was prepared as 0.5 g collagen in 50 mL of deionised H_2_O, pH 3.2. Microbial transglutaminase (mTG) (a gift as maltodextrin, Ajinomoto^®^) was purified using the AKTA system (GE Healthcare, Little Chalfont, UK), following a method adapted from Kuwahara et al. 2011 [5].

### Scaffold fabrication

For collagen fibrillogenesis, collagen solution and sodium phosphate buffer (50 mM sodium dihydrogen phosphate, 10 mM potassium dihydrogen phosphate) were mixed in 1:1 ratio and incubated at 37 °C overnight [6–8]. Afibrillar and fibrillar collagen scaffolds were then frozen at −20 °C for 24 h and freeze-dried under a vacuum of 5 Pa (Christ I-5, Martin Christ Gefriertrocknungsanlagen GmbH, Osterode am Harz, Germany).

Collagen /nano-hydroxyapatite [HA]/chondroitin sulphate [CS] scaffolds were prepared as for collagen scaffolds with the addition to the collagen solution at the time of blending, of nanoHA (30, 50 and 75 wt%) (Sigma-Aldrich, St Louis, MO, USA) and CS (1 wt%) (Sigma-Aldrich). Collagen/mTG scaffolds were fabricated as for collagen scaffolds with the addition of 300 U mTG per gram of collagen during fibrillogenesis, pH 7.4.

### Scaffold characterisation

High-resolution scanning electron microscopy (SEM) was performed with a Jeol scanning microscope JSM840F (Jeol Ltd, Tokyo, Japan), with a working distance of 10–30 mm, electron voltage of 3–5 kV and 50–25,000 × magnification. Cylindrical scaffold samples, 10 mm in diameter, were sputter-coated with 3 nm platinum (280HR sputter coater, Cressington Scientific Instruments Ltd, Watford, UK) and mounted onto aluminium stubs with adhesive carbon tabs.

SEM images were analysed with ImageJ software to calculate pore size, and the result was calculated as the mean of 3 individual areas per scaffold [9–11].

Transmission electron microscopy (TEM, using a 2000FX, Jeol Ltd) was used to visualise the D-banding pattern of collagen and any changes in the ultrastructure of the collagen scaffolds after fabrication. Collagen samples were re-suspended in distilled water, placed on copper TEM grids (Agar Scientific, Stansted, UK), air-dried overnight and then examined using bright field imaging mode at an accelerating voltage of 200 kV.

Using a PerkinElmer Hyper Differential Scanning Calorimeter, 5–10 mg of each scaffold was placed in a 40-μl aluminium pan (PerkinElmer) and heated to 180 °C at 50 °C min^-1^. The raw data were compiled and analysed using Pyris™ Data Analysis Software (PerkinElmer, Waltham, MA, USA), recording the denaturation temperature for each sample.

Thermogravimetric analysis (TGA) was performed with 5–10 mg sample heated from 37 to 900 C at a rate of 10 °C min^−1^ in a nitrogen atmosphere purged at 100 mL min^−1^ (Diamond TG/DTA, PerkinElmer, Waltham, MA, USA). Atomic force microscopy (AFM) was performed with 1 cm^2^ samples mounted on a Park Instruments (Suwon, Korea) AutoProbe Atomic Force Microscope, scanned in non-contact mode using Ultrasharp NSC12 diving-board levers with spring constants of 4–14 N m^−1^ and resonant frequencies of 150–310 kHz.

The homogeneity of the mineralised collagen scaffold was analysed by SEM using a Jeol 6480 LV electron microscope with energy-dispersive x-ray (EDX), mapped with respect to Ca^2+^, P^5+^ and O^2-^ ions (three regions per sample, 15 kV, 40 Pa with backscattered contrast and 100 × magnification).

X-ray diffraction (XRD) analysis was performed using a fully automated Bruker D5000 powder diffractometer employing copper kα radiation (λ = 0.15406 nm) and a secondary monochromator, using a step size of 0.05°2θ from 10–70°2θ with a count time of 15 s step^-1^.

Young’s modulus of the mineralised collagen scaffold was evaluated by uni-directional compression testing of samples (n = 3) 12.5 mm in diameter and 13.5–15 mm in height (PerkinElmer Dynamic Mechanical Analyzer 7e) [12].

### Stem cell culture and seeding

Human mesenchymal stem cells (hMSC) (Lonza, Walkersville, MD, USA) were grown in MSCGM™ (Mesenchymal Stem Cell Growth Medium) (Lonza) supplemented with BulletKit^™^ (Lonza). Cells were maintained on Matrigel™-coated plates (Matrigel™, BDBiosciences, Franklin Lakes, NJ, USA #354262) in a humidified incubator with 5% CO_2_ at 37 °C. Passage was performed at 70% confluence using 0.05% Trypsin EDTA (Invitrogen, Carlsbad, CA, USA).

### Cell growth and viability assays

All experiments were performed with biological and technical triplicates. Cylindrical collagen scaffolds used for hMSC culture measured 8 mm diameter, 3 mm height and were sterilised using gamma irradiation (10 KGy, GSR D1 irradiator, Gamma-Service Medical GmbH, Leipzig, Germany). Cells were seeded at a density of 1 × 10^5^ cells per scaffold, cultured as for maintenance and harvested using collagenase type I (Sigma #CO130, 100 collagenase digestion units per scaffold, 1-h incubation at 37 °C).

Quantitative assessment of viable cell number over time, and hence proliferation rate, was performed using an AlamarBlue^®^ assay (Life Technologies #DAL1025). After a brief vortex, AlamarBlue^®^ was added to the cells at 10% of total volume. After 2-h incubation at 37 °C and a brief vortex, fluorescence of the medium/AlamarBlue was read at an excitation wavelength of 570 nm and emission of 585 nm (POLARstar Omega, BMG Labtech, Ortenberg, Germany).

Three-dimensional fluorescent live cell imaging of MSCs within the collagen-based scaffolds was performed by multiphoton microscopy (MPM) using a modified Bio-Rad Radiance 2100 MP Multiphoton Microscope (Institute of Biomedical Engineering, Oxford, UK). Near-infrared (NIR) laser beams (wavelength 800 nm) were produced by a tunable 76 MHz femtosecond pulsed Ti:sapphire laser (Mira 900-F, Coherent, Ely, UK) pumped by a 10 W multiline argon ion laser (Verdi; Coherent). A Nikon S Fluor 20 × objective with a numerical aperture of 0.75 was used for all images. The emission filters selected were: 495 nm for blue, 525 nm for green and 595 nm for red. Scanning speed was 166 lines per second, and the Z-step was 2.55 μm. Imaris 7.6.1 (Bitplane, Zurich, Switzerland) was used for image processing.

### Osteogenic differentiation

The expression of critical osteogenic genes in scaffold-grown MSCs was quantitatively analysed using RT-PCR. Osterix (Sp7), osteocalcin (BGLAP), ALP, BMP-2, TGFβl and RUNX2 were selected for analysis based on their previous identification as markers of osteogenesis [13–19].

After 3 days, MSCs were harvested from scaffolds by collagenase digestion at 37 °C for 1 h followed by centrifugation at 3,000 rpm for 3 min at RT. RNA was extracted using TRI^®^ Reagent (Sigma) according to the Sanger Institute Protocol (http://www.sanger.ac.uk/Projects/Microarrays/arraylab/protocoll.pdf) and quantified using a NanoDrop 2000 Spectrophotometer (Thermo Fisher Scientific, Waltham, MA, USA).

cDNA was synthesised using high capacity RNA-to-cDNA MasterMix™ (Applied Biosystems, Foster City, CA, USA #4390779) according to the manufacturer’s protocol, using 2 pg RNA in a 20 pL reaction volume.

RT-PCRs were performed using 5 pL SYBR® Green PCR Master Mix™ (Applied Biosystems #4385618), 400 nM of forward and reverse primers and 1 pL of cDNA in a total volume of 10 pL (made up using RNAase-free water), using technical and biological triplicates. Cycling conditions were 2 min at 50 °C, 10 min at 95 °C and 40 cycles of 15 s at 95 °C followed by 1 min at 60 °C, 15 s at 95 °C, 15 s at 60 °C and a final 15 sec at 95 °C. All reactions were performed on an Applied Biosystems 7500 Fast Real-Time PCR System, and data were normalised to a panel of housekeeping genes (β-actin, UBC, TBP and GAPDH) using the comparative Ct method, where ‘reference gene’ denotes the average Ct value across the panel of housekeeping genes [20, 21]. All primers were designed using PrimerQuest (http://eu.idtdna.com/PrimerQuest), and sequences are shown in Supplementary Material 1.

## Results

### Imaging

**Figure 1:**
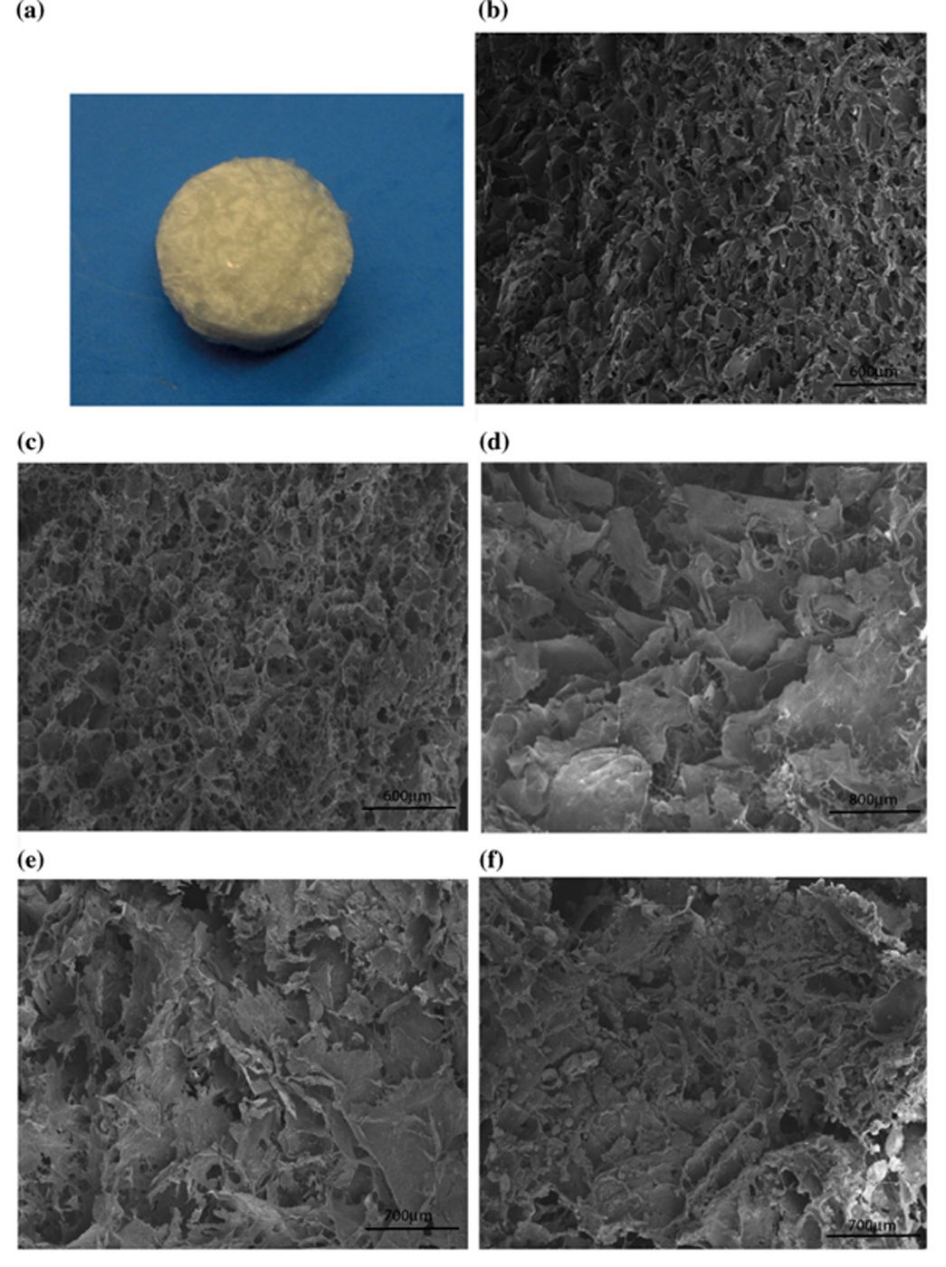
Scanning electron microscopy images show porous structure of cut surface of collagen and collagen/CS/HA scaffolds **a** Photograph of fibrillar collagen scaffold. SEM images of **b** afibrillar collagen, **c** fibrillar collagen, **d** collagen/CS 1wt%/HA 30wt%, **e** collagen/CS 1wt%/HA 50wt%, **f** collagen/CS 1wt%/HA 75wt%. All images were obtained using a JEOL scanning microscope 840F with a working distance of 10–30 mm, electron voltage of 5 kV and 50–10,000× magnification. CS chondroitin sulphate; HA hydroxyapatite.

**Figure 2:**
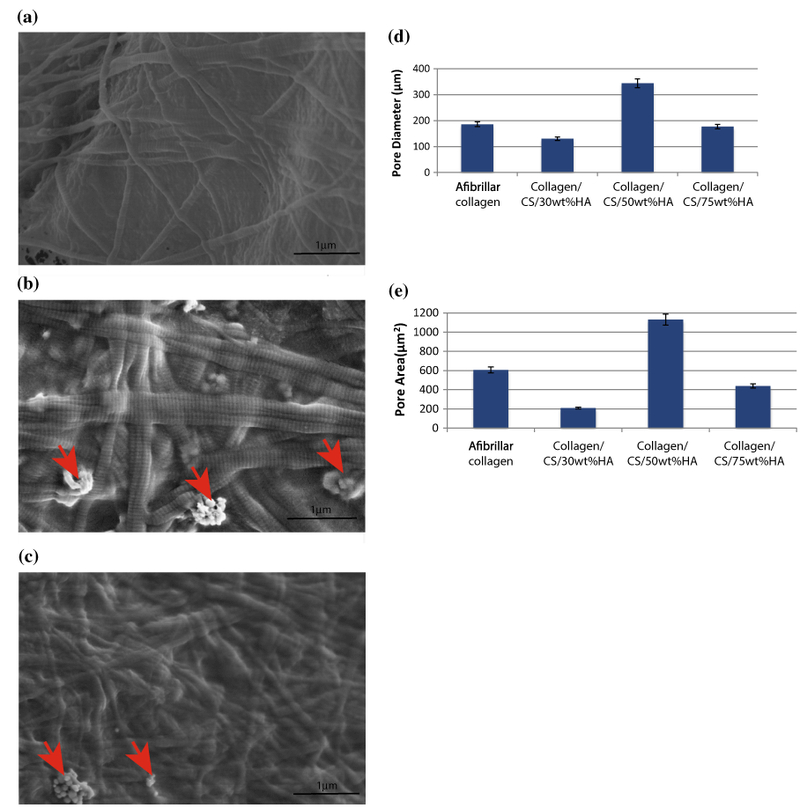
SEM images show D-bandmg spacing of collagen, with agglomerates of nanoHA interwoven throughout the fibrils and calculated pore size **a** Fibrillar collagen, **b** collagen/CS 1wt%/HA 50wt%, **c** collagen/CS 1wt%/HA 75wt%. *Graphs* represent average pore diameter (**d**) and area (**e**) of afibrillar and HA/CS collagen scaffolds. Experiments performed in triplicate; *error bars* show standard deviation. All SEM images were obtained using a JEOL scanning microscope 840F, working distance of 10–30 mm, electron voltage of 3–5 kV and 25,000× magnification. ImageJ software was used to calculate pore size of collagen scaffolds from SEM images. *CS* chondroitin sulphate; *HA* hydroxyapatite. *Arrowhead,* nanoHA.

The collagen scaffold (Fig. 1a) was cut and imaged using SEM. Both afibrillar and fibrillar collagen contained pores, which were uniformly distributed (Fig. 1b, c). A homogeneous pore microstructure with interconnected polyhedral pores was observed throughout the scaffolds (Fig. 1b-f). The cut surfaces of fibrillary collagen/CS/HA scaffolds containing an increasing wt% of HA showed that the interconnecting pore structures were maintained (Fig. 1d, f). At higher magnifications, self-assembling fibres with characteristic D-banding spacing distribution of fibrillar collagen and nanoparticles of HA with agglomeration were observed (Fig. 2a-c). The mean pore diameter and area within all the scaffolds were calculated to be within the ranges 130–186 μm and 200–600 μm^2^, respectively, apart from collagen/CS/50 wt% HA which had larger pores (Fig. 2d-e).

**Figure 3:**
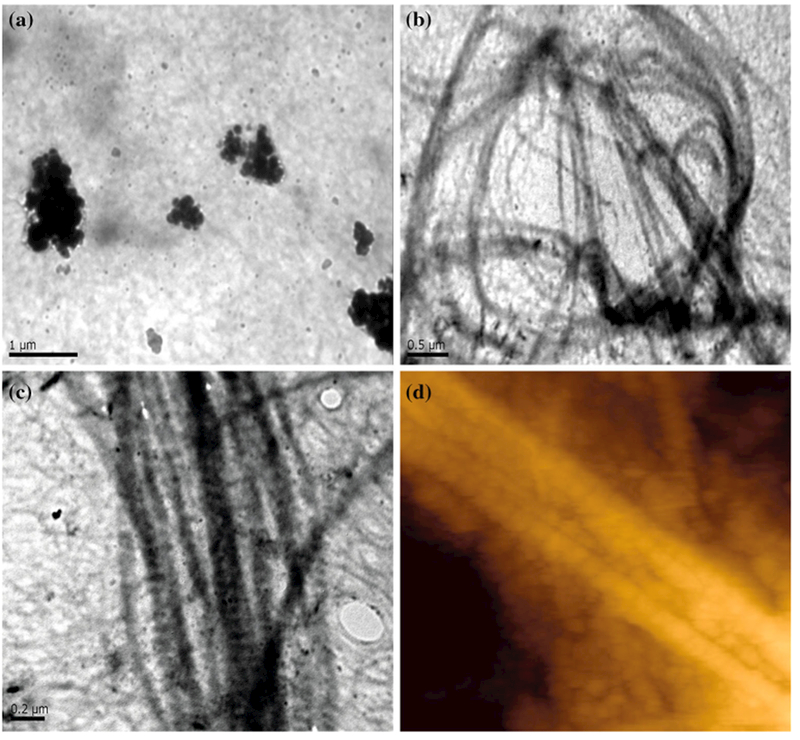
Transmission electron microscopy (TEM) and atomic force microscopy (AFM) images show D-banding spacing of collagen, with agglomerates of nanoHA interwoven throughout the fibrils. TEM images of **a** afibrillar collagen, **b** and **c** fibrillary collagen/CS 1wt%/HA 50wt%, scale as shown. **d** AFM image of fibrillar collagen scaffold. CS chondroitin sulphate; HA hydroxyapatite.

TEM supported the findings of SEM, revealing the characteristic D-banding patterns of fibrillar collagen observed in collagen/CS/HA scaffolds, which were not present in afibrillar collagen (Fig. 3a-c). Collagen scaffolds were scanned using AFM and non-contact mode images confirmed the ultrastructural D-banding pattern within the collagen fibrils (Fig. 3d).

### Chemical composition

**Figure 4:**
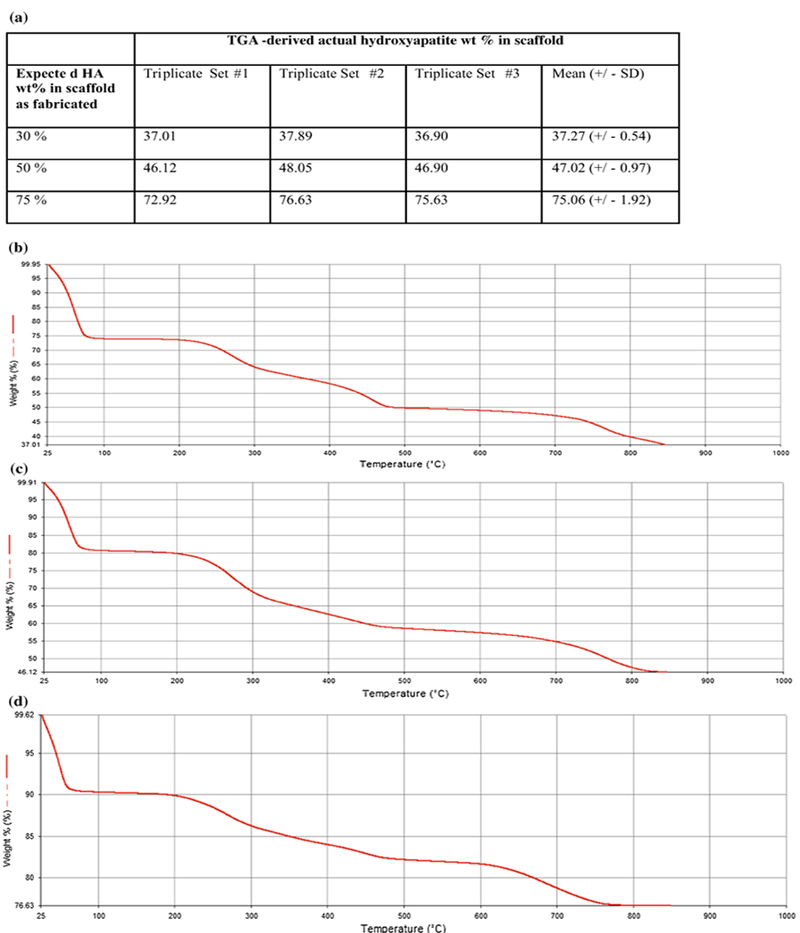
Thermogravimetric analysis (TGA) confirms composition of scaffolds. **a** Summary table of TGA-derived wt% values of HA in collagen/CS/HA scaffolds. Data are shown for each sample in triplicate with mean values ± SD. TGA data for scaffolds as prepared with 30–75 wt% HA and heated from 25 to 1000 ^0^C at a rate of 100Cmin^−1^ in nitrogen. *Graphs* show representative data for each scaffold prepared with desired weight percentage of HA **b** 30 **c** 50 and **d** 75. CS chondroitin sulphate; HA hydroxyapatite.

TGA showed differences between the expected and actual weight per cent of HA within the scaffolds (Fig. 4a). Overall, TGA confirmed the content of an increasing wt% of HA as fabricated within collagen/CS/HA scaffolds (as expected). The thermograms of each of these scaffolds all showed the same pattern (Fig. 4b-d): up to 200 °C the absorbed water was evaporated; subsequent loss in weight of collagen molecules was observed from 200 to 500 °C; a slight loss was observed from 500 to 700 C due to further combustion of residual collagen/CS components.

**Figure 5:**
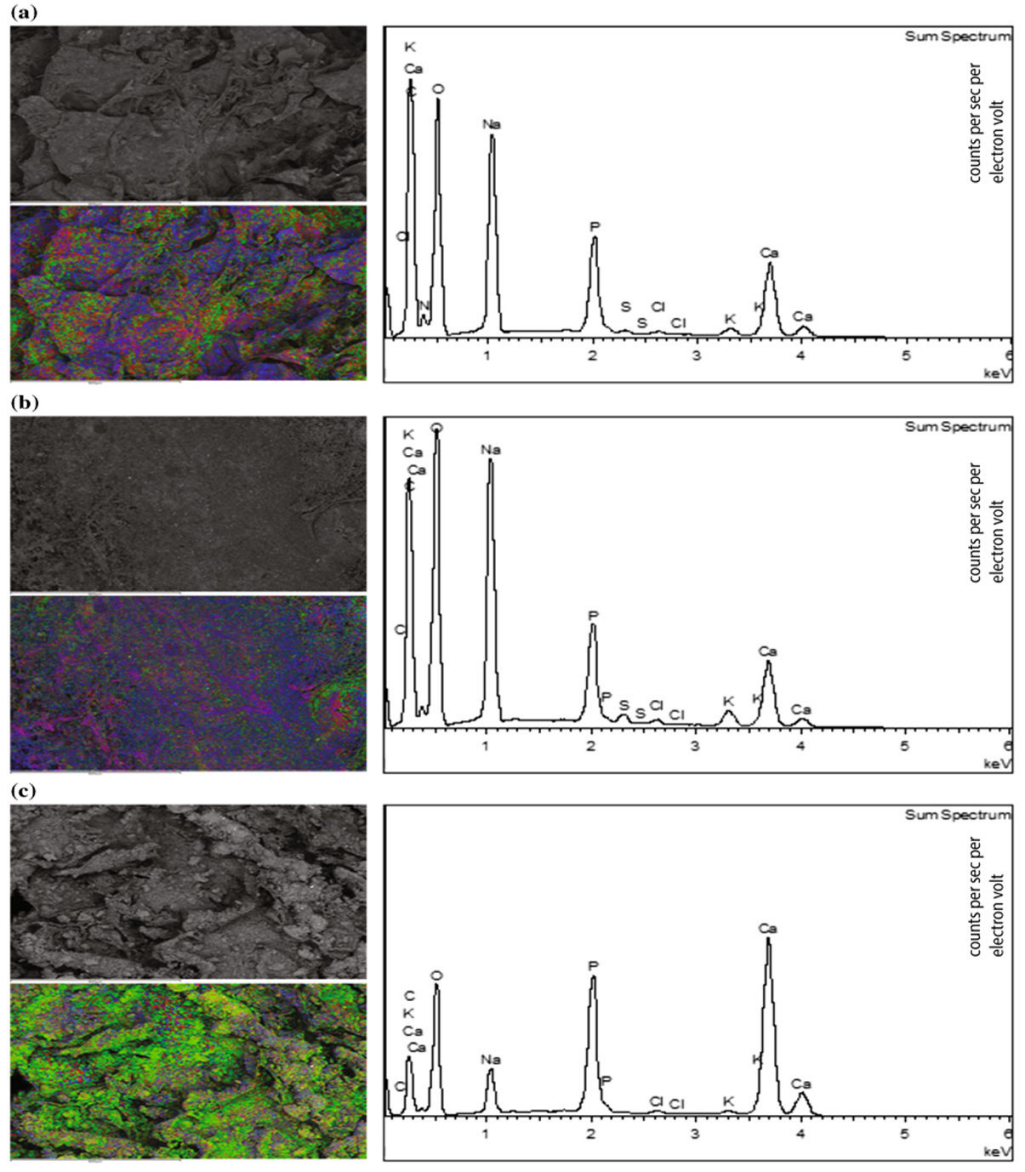
Energy dispersive x-ray (EDX) analysis of collagen scaffold with increasing wt% of hydroxyapatite (HA). Collagen scaffold containing **a** 30 wt% **b** 50 wt% and **c** 75 wt% HA was analysed by EDX. For each sample, SEM images are shown for the cross-sectional cut surface with and without EDX overlay, demonstrating the homogeneity of HA throughout the scaffold. *Red,* phosphorous; *Green,* calcium; *Blue,* oxygen. For each sample, graphs of elemental mapping are shown on the *right. HA* hydroxyapatite; *SEM* scanning electron microscopy.

EDX analysis of collagen scaffolds with increasing wt% HA showed homogenous distribution of HA throughout the scaffolds (Fig. 5).

**Figure 6:**
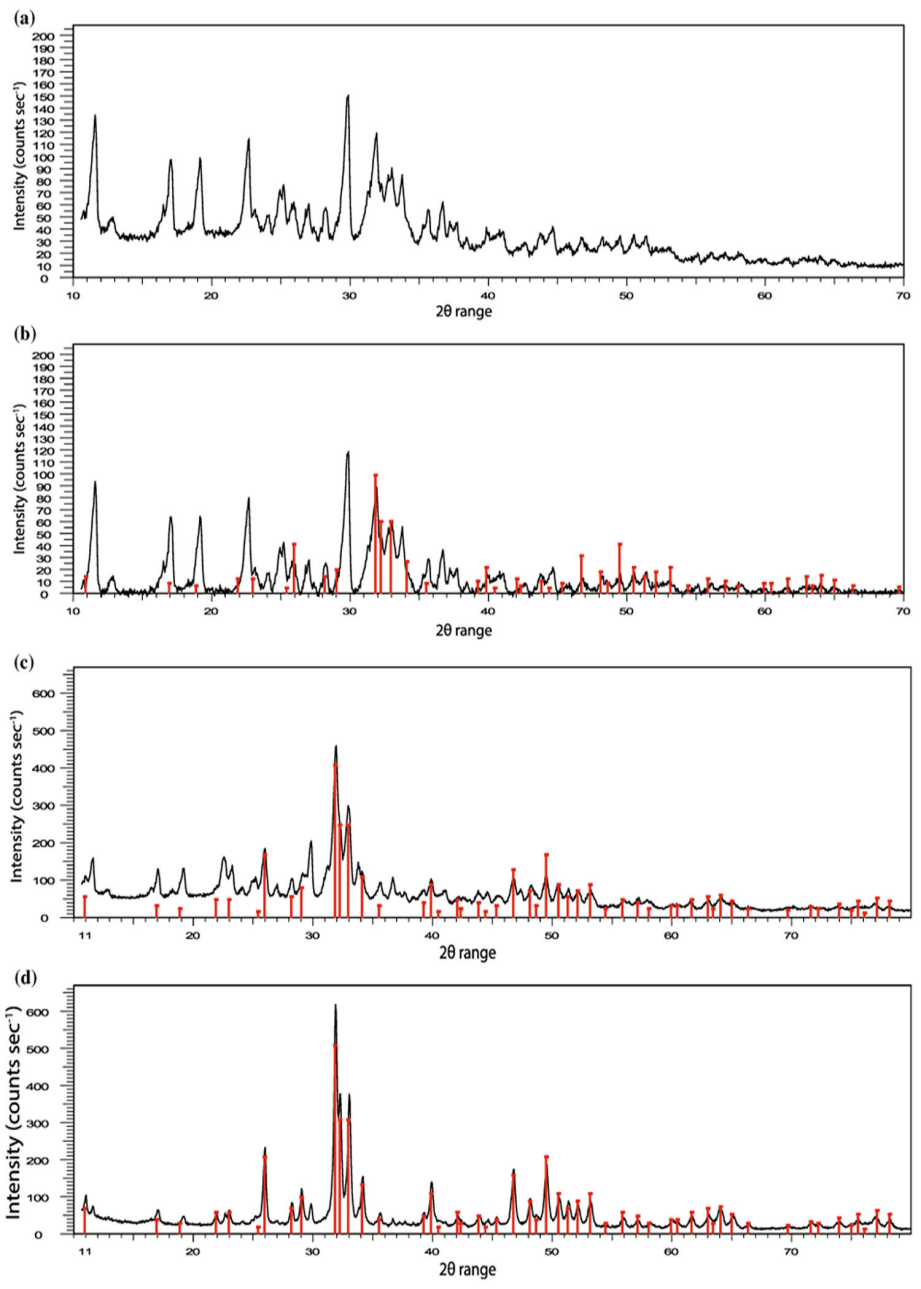
X-ray diffraction (XRD) analysis of collagen scaffolds XRD was performed over a 2_ range of 10–800 with a step size of 0.05, dwell time of 12 s at RT and divergent slit of 0.5. Bruker analysis software was used to analyse the data. **a** ICDD standard diffraction pattern for HA (#09–432). XRD plots for collagen/CS scaffolds containing **b** 30 wt%, **c** 50 wt% and **d** 75 wt% HA. RT room temperature; XRD X-ray diffraction; *ICDD* International Centre for Diffraction Data; CS, chondroitin sulphate; *HA* hydroxyapatite.

XRD patterns were analysed using the International Centre for Diffraction Data (ICDD) standard diffraction pattern for HA (#09–432, Fig. 6a). XRD patterns were obtained for collagen/CS/HA scaffolds containing 30, 50 and 75 wt% HA (Fig. 6b-d) and the samples were found to be exact matches for HA, confirming the phase purity of the HA and its homogeneous distribution throughout the scaffolds.

### Thermal analysis

**Figure 7:**
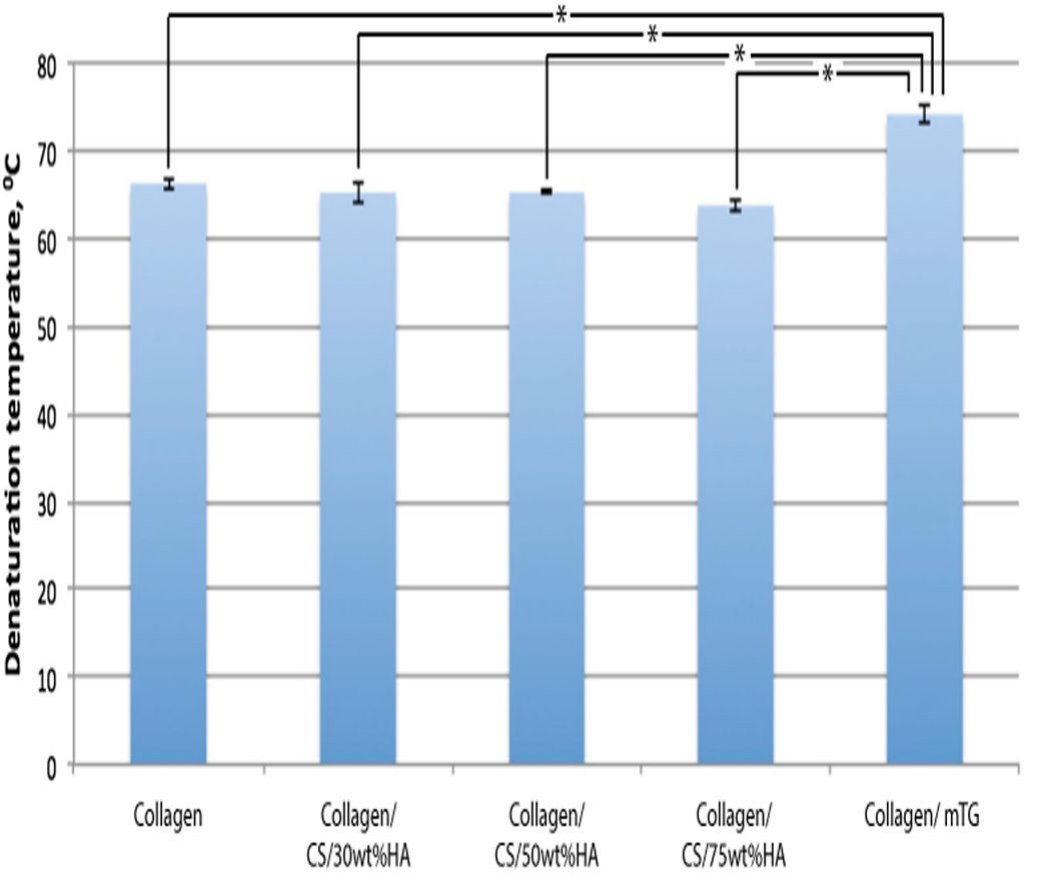
Dynamic scanning calorimetry (DSC) shows thermal stability of scaffolds at body temperature. Graph of mean denaturation temperatures for each scaffold type, calculated from DSC data of peak temperature. *Error bars* standard deviation; *P < 0.02. CS chondroitin sulphate; *HA* hydroxyapatite.

DSC was used to assess the thermal stability of the scaffolds, and thus their suitability for in vivo use. Denaturation temperatures were similar for all samples tested (average 65.1 °C, range 63.7–66.2 °C), apart from mTG-cross-linked collagen, which had a significantly higher Td of 74.1 °C (one-way analysis of variance, *P <* 0.02, Fig. 7).

### Mechanical characterisation

**Figure 8:**
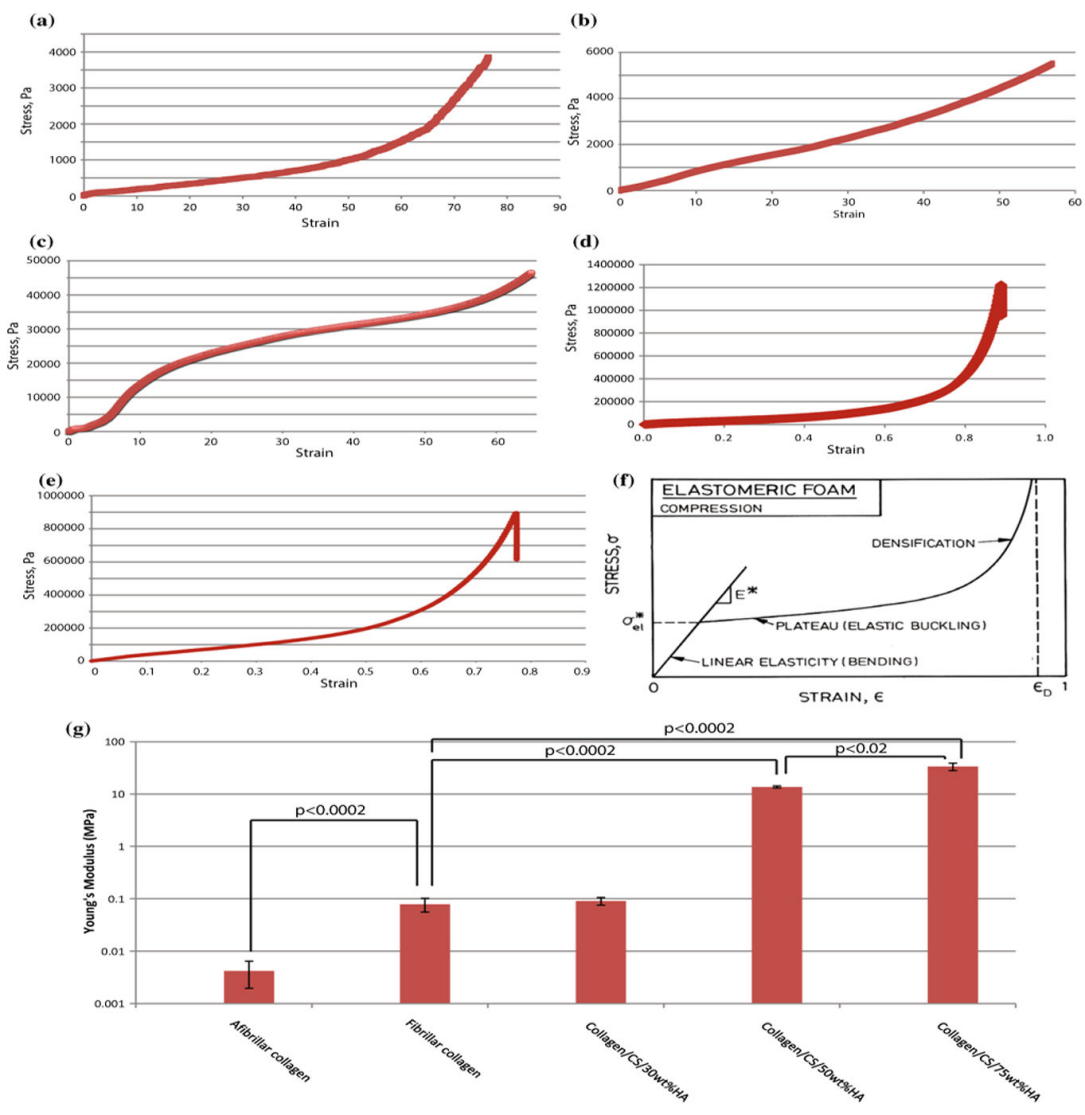
Mechanical testing of collagen scaffolds Compression testing was performed. *Graphs* showing stress/strain curves for **a** afibrillar collagen, **b** fibrillar collagen and scaffolds containing collagen/CS with **c** 30, **d** 50 and **e** 75 wt% HA. **f** Schematic representation of calculation of Young’ modulus from stress/strain curves, Gibson, 2000. **g** Graph of Young’s moduli calculated using LINEST function in Microsoft Excel; log scale, average of triplicate samples. CS chondroitin sulphate; HA hydroxyapatite.

Mechanical testing was performed to establish the compressive modulus for the scaffolds. Only compressive strength was evaluated, as the scaffold is intended for application to spinal surgery models, where the principal force is compression. Representative stress/strain curves for each sample tested are shown (Fig. 8a-e).

From such curves, Young’s modulus was calculated as shown in the schematic representation in Fig. 8f, using a curve with three distinct parts: linear elastic, collapse plateau, densification [12]. The average values of triplicate samples for each scaffold are shown (Fig. 8g). Afibrillar collagen had the lowest Young’s modulus of all samples tested (0.004 MPa, Fig. 8g), which was significantly lower than that of fibrillar collagen (0.079 MPa, *P <* 0.0002, Student’s *t* test). The addition of 30 wt% HA did not significantly change the compressive modulus (0.079 MPa). However, at higher HA wt% (50 and 75), there was a large, highly significant increase in compressive modulus (13.7 and 33.6 MPa, respectively, *P <* 0.0002 for both).

### Cell distribution and growth

**Figure 9:**
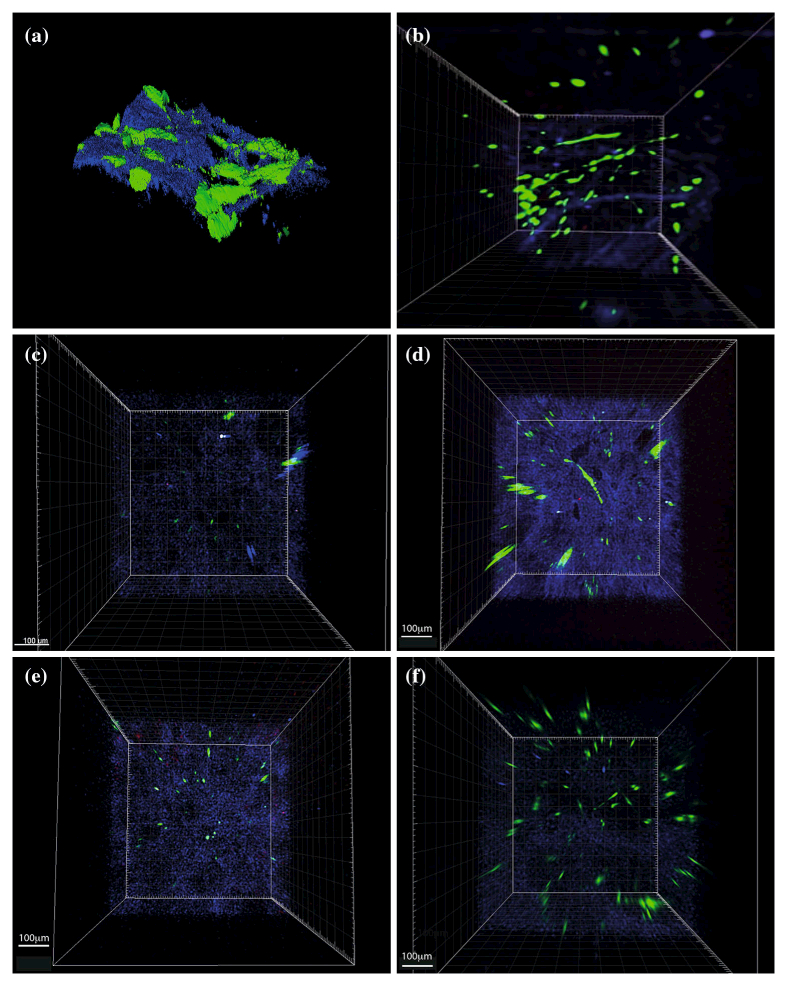
Viable human mesenchymal stem cells grow throughout 3-dimensional collagen-based scaffolds. **a** 3-D reconstruction of hMSCs in collagen scaffold. Representative multiphoton microscopy images of live hMSCs in **b** collagen, **c** collagen/CS/ HA30wt%, **d** collagen/CS/HA50wt%, **e** collagen/CS/HA75wt% and **f** collagen/mTG scaffolds. 100,000 hMSCs seeded per scaffold, all images obtained after 3 days of culture using a BioRad Radiance 2100 MP Multiphoton Microscope. *Blue* collagen; *Green* live cells; *Red* dead cells.

After allowing the human mesenchymal stem cells (hMSCs) to grow for 3 days, viable cells were observed by MPM and were evenly distributed throughout each of the collagen-based scaffolds (representative images shown in Fig. 9a-f).

**Figure 10:**
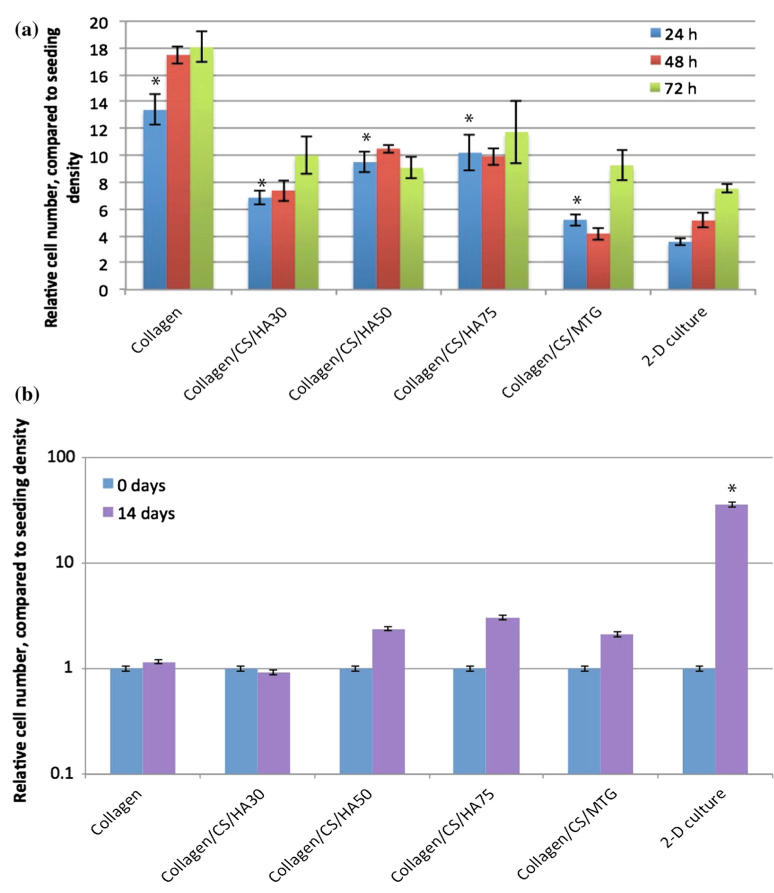
Collagen-based scaffolds support the growth and differentiation of human mesenchymal stem cells. **a** *Graph* showing relative number of hMSCs grown on collagen, collagen/CS/ HA30 wt%, collagen/CS/ HA50wt%, collagen/CS/ HA75 wt%, collagen/CS/ MTG or 2-D culture, compared to seeding density, using Alamar Blue assay. Values at 24, 48 and 72 h. **b** *Graph* showing relative number (shown as log scale) of hMSCs grown on collagen, collagen/CS/HA30 wt%, collagen/CS/HA50 wt%, collagen/CS/HA75 wt%, collagen/CS/MTG or 2-D culture, compared to seeding density, using Alamar *Blue* assay. Values at 0 and 14 days. Mean values, error *bars* represent standard deviation. *P < 0.05, unpaired *t* test with Welch’s correction.

Three-dimensional collagen scaffolds promoted significantly more cell proliferation than 2-D Matrigel^™^-coated plastic culture plates (*P <* 0.02, Fig. 10). Over time, although the cells continued to grow in the scaffolds, there was a relative reduction in the rate of increase in cell number, compared to cells in 2-D culture (Fig. 10a-b). At 72 h, there was no significant further increase in cell number for hMSCs grown in HA-containing collagen scaffolds, whilst hMSCs grown in 2-D culture, and those in collagen/CS/mTG and collagen scaffolds did continue to proliferate. By 14 days, the cells grown in 2-D culture had increased in number significantly more than the cells grown in any of the collagen scaffolds (Fig. 10b).

### Cell differentiation

**Figure 11:**
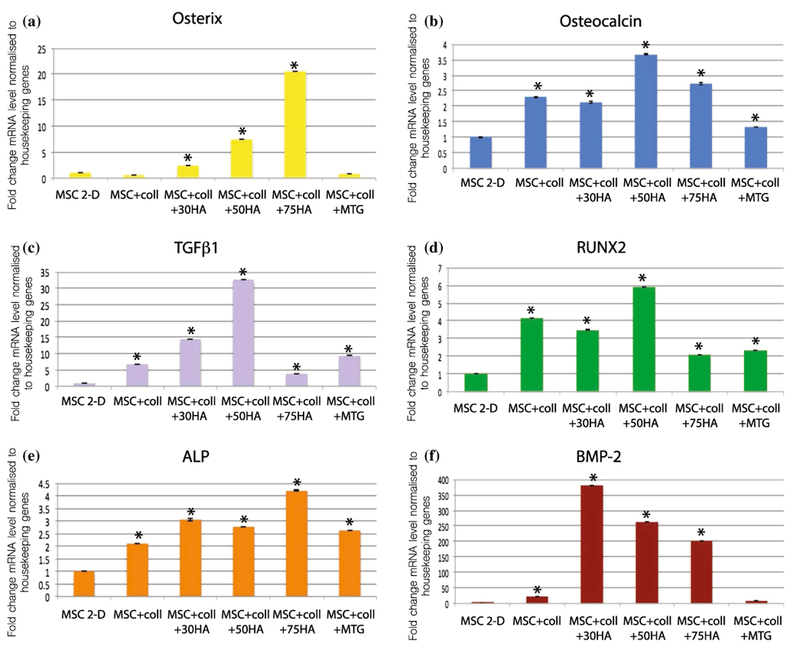
Osteogenic gene expression is enhanced by the presence of collagen-based scaffolds. Quantitative gene expression in hMSCs harvested from either 2-D culture on Matrigel-coated dishes or 3-D collagen-based scaffolds (collagen, collagen/CS/ 30 wt% HA, collagen/CS/50 wt% HA, collagen/CS/75 wt% HA, collagen/mTG) using RT-PCR. Values shown for fold change in expression level of mRNA normalised to a panel of housekeeping genes (TBP, β-ACTIN), *error bars* represent standard deviation. Genes tested were **a** osterix, **b** osteocalcin, **c** TGF-βΙ, **d** RUNX2, **e** alkaline phosphatase, **f** BMP-2. *P < 0.05, unpaired t test using Welch’s correction.

Quantitative gene expression showed a significant increase in the mRNA levels of osterix in hMSCs grown in collagen/CS/HA scaffolds compared to cells in 2-D culture or in collagen-only scaffolds, with the greatest increases seen in collagen/CS/HA 50wt% and collagen/CS/HA 75wt% scaffolds which exhibited a 7.5- and 20.6-fold increase, respectively, relative to 2-D culture and normalised to housekeeping genes (*P <* 0.05, Fig. 11a).

Expression levels of osteocalcin, TGFβ1 and RUNX2 were significantly higher in cells grown in collagen-based scaffolds than in 2-D culture with the greatest increase observed in collagen/CS/HA 50wt% scaffolds (fold change of 13.9, 32.7 and 22.2, respectively, *P <* 0.05, Fig. 11b-d). Significantly higher expression of ALP was observed in cells grown in collagen scaffolds compared to cells grown in 2-D culture, with the highest levels seen in collagen/CS/HA 75wt% scaffolds (fold change of 4.2, *P <* 0.05, Fig. 11e). Expression levels of BMP-2 were significantly higher in cells grown on all scaffolds apart from collagen/CS/mTG, compared to cells in 2-D culture, with the highest levels observed in cells from collagen/CS/HA 30wt% and collagen/CS/HA 50wt% scaffolds (fold changes of 1025.2 and 994.0, respectively, *P <* 0.05, Fig. 11f).

**Figure 12:**
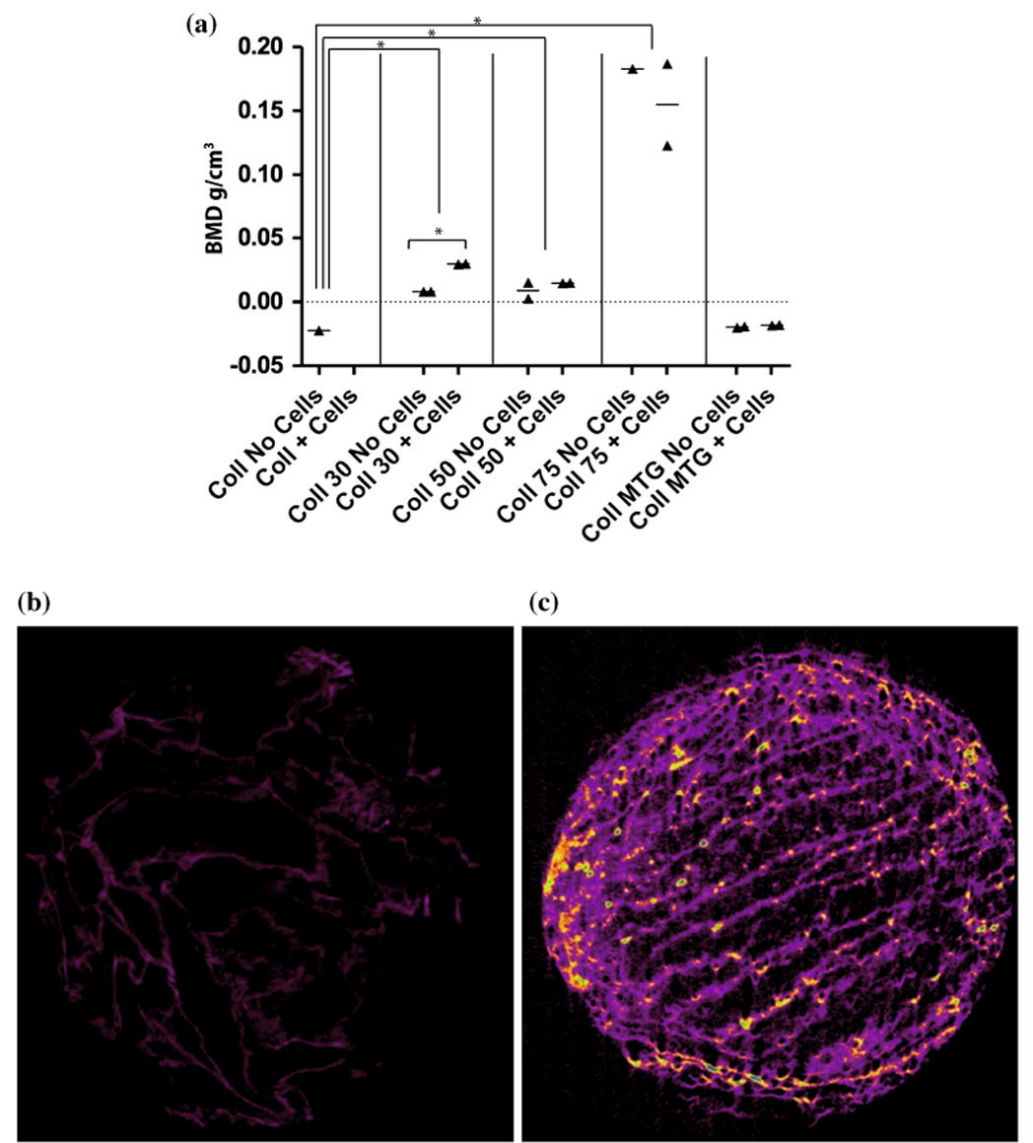
Bone mineral density of collagen scaffolds is increased with the presence of HA. **a** *Graph* showing mean BMD of collagen scaffolds with and without hMSCs, after 7 days of culture. *Error bars* standard deviation. *P < 0.02. Representative micro-CT images of **b** collagen and **c** collagen/75wt% HA scaffolds, obtained using Skyscan 1174 (Bruker, Belgium) compact micro-CT scanner, at 50 kV, 800 μA.

Micro-CT revealed a significant increase in BMD with the addition of HA to the collagen scaffolds (*P <* 0.02, Fig. 12a). Culture of hMSCs on collagen/30wt% HA scaffolds resulted in a significant increase in BMD (*P <* 0.02, Fig. 12a) compared to collagen-only scaffolds. Representative images obtained from micro-CT analysis are shown in Fig. 12b-c.

## Discussion

We have successfully fabricated a novel collagen-based scaffold and characterised it using a range of imaging, chemical composition and thermal analysis techniques. Self-organising atelocollagen was combined with nano-hydroxyapatite and chondroitin sulphate, and the effect of cross-linking was studied using microbial transglutaminase. Hydroxyapatite has been widely used as an effective bone substitute material since the 1980s, as it provides a stable interface between itself and bone, the ‘apatite layer’ [22]. Recently, interest has grown in the potential use of HA nanoparticles since they have a greater surface area and more active free sites than microparticles, leading to improved bone healing [23, 24]. However, HA alone lacks mechanical strength, limiting its usefulness as a graft for clinical application. Chondroitin sulphate, the most abundant glycosaminoglycan in the human body, was added to the scaffold as it is a normal constituent of bone and has been shown to minimise the inflammatory-mediated catabolism of cartilage and bone as well as to promote bone anabolism [25].

The ultrastructure of collagen is critical to its biological functions, including cell adhesion. The pH and ionic concentration of the buffer used for fibrillogenesis has a significant effect on the formation of classical D-banded fibrils. Detailed studies have shown that optimal fibrillogenesis requires near-physiological conditions, with a pH of 7.4 and low ionic strength (below 150 mM) [7, 26]. In our fibrillogenesis process, we used a buffer of pH 7.4 and ionic strength 60 mM, which is similar to the conditions used in previous studies. We then used a range of imaging techniques, including SEM and TEM, and demonstrated that self-organising collagen forms fibrils, which exhibit the characteristic D-banding pattern.

Our results reveal the presence of single collagen fibrils in the scaffold, interspersed with HA, as found in normal human bone. The presence and homogeneous distribution of HA was confirmed using XRD and EDX. The porous structure of scaffolds after freeze-drying arises from the deposition of ice crystals within the collagen solution, which then evaporate under vacuum. The pore size of scaffolds in tissue engineering is critical for cell growth and function and is cell-type specific [27]. As osteoblasts are 20–30 μm in diameter, studies have shown that for bone tissue engineering, the minimum pore diameter is approximately 100 μm [28]. Moreover, pore sizes greater than 300 μm diameter have been shown to enhance cell migration, osteogenesis and capillary formation [29]. Our scaffolds were found to contain evenly distributed pores with a diameter appropriate to allow cell migration and promote osteogenesis.

DSC was used to study the thermal stability of all samples at physiological body temperature, 37 °C. Interestingly, the presence of mTG led to a significant increase in thermal stability, with a denaturation temperature of 74 °C. Although a small amount of cross-linking normally occurs during fibrillogenesis, addition of mTG to the collagen solution was able to promote cross-linking of collagen fibrils in the scaffolds. Such increased thermal stability suggests a slower degradation profile of collagen/mTG scaffolds and therefore the potential for a longer-lasting scaffold in vivo. Similar results have been obtained for type II collagen scaffolds, with mTG decreasing the rate of degradation [30]. Such a degradation profile would enable the scaffold to promote bone growth over a longer period of time, which could in turn promote fusion rates.

mTG catalyses the formation of covalent bonds between a free amine group from a protein (for example, collagen) or peptide-bound lysine and the γ-carboxamide group of a protein (for example, collagen) or peptide-bound glutamine [31]. Other crosslinking agents have previously been studied in scaffolds for bone regeneration, including glutaraldehyde, EDC (1-ethyl-3-(3–3-dimethylaminopropyl) carbodiimide hydrochloride), NHS (N-hydroxysuccinimide) and UV radiation, due to the improvement in mechanical and physicochemical properties of collagen scaffolds observed with their use. However, disadvantages of these techniques include toxicity and altered structural properties of the material. mTG, an enzymatic cross-linking agent, has recently gained interest for in vivo use due to its lower toxicity compared to chemical cross-linking agents, and it was therefore chosen for this work with potential clinical application.

Mechanical testing of the fabricated scaffolds showed distinct linear elastic, collapse plateau and densification regions, and the Young’s modulus of mineralised collagen (50 and 75 wt% HA, 13.7 and 33.6 MPa, respectively) was well above that of developing osteoid tissue (25–100 kPa) and close to that of cancellous bone (90–400 MPa); therefore, it should provide an appropriate stiffness for the adhesion, growth and differentiation of MSCs [32–34].

When human MSCs were seeded onto the scaffolds, they showed good penetration of the graft material and by 72 h were evenly distributed throughout all the 3-D scaffolds. During the initial phase of culture, cell proliferation was accelerated in the 3-D scaffolds compared to 2-D culture, but then slowed, suggesting that the MSCs may have switched from proliferation to differentiation. This was supported by increased expression of osteogenic marker genes in the scaffold-grown cells compared to 2-D culture. Cells grown on collagen/30wt% HA scaffolds also showed increased BMD of the matrix compared to scaffolds without HA.

Based on our results, collagen/CS/HA 75wt% scaffolds show the most optimal properties for further studies before in vivo application, including a large pore size and good compressive strength. In addition, collagen/CS/HA 75wt% scaffolds show the best ability to induce bone formation with an increase in osteogenic gene expression of hMSCs, particularly with respect to the levels of BMP2, osteocalcin, RUNX2 and TGFβ1.

Currently available clinical graft materials include demineralised bone matrix, collagen, ceramics and BMPs (using a carrier material such as HA, with MSCs or collagen). Only a few ‘combination’ grafts are approved for use in patients undergoing spinal surgery, such as Infuse^®^ (Medtronic, USA; recombinant human BMP-2 and collagen) and OP-1 (Stryker, USA; recombinant human BMP-7 and collagen). The BMP/collagen grafts are not available as a composite scaffold, rather mixed together before application as a graft, and they lack significant mechanical strength. Furthermore, the long-term safety profile of recombinant human BMPs is poorly understood and remains a concern. Currently in the UK, only two bone substitutes have level I data for spinal surgery (Vitoss, Stryker (β-TCP/collagen) and Cortoss, Stryker (bioactive glass cement). Cortoss has a smaller pore size than of our scaffolds, at approximately 150 μm, and a high compressive strength (40–50 MPa) [35]. Whilst both these scaffolds have been shown to possess osteoconductive properties, neither of these contains chondroitin sulphate, an important osteogenic glycosaminoglycan present in normal human bone. Another limitation is the brittleness of bioactive glass.

Whilst all clinically approved grafts have some strengths, a common limitation is the inability to combine all the ideal properties of a graft. We have shown our scaffold to display osteoconductive, osteogenic and osteoinductive properties, and therefore, we believe this scaffold will be a superior graft. Further studies could include a direct comparison with our scaffold and other commercially available scaffolds, both in vitro and in vivo.

## Conclusions

We have developed a novel bone graft biomaterial prepared from natural constituents of bone, which shows good mechanical and thermal characteristics and promotes the proliferation and then the osteogenic differentiation of mesenchymal stem cells. This suggests that our scaffold material combined with hMSCs shows promise for clinical application in spinal fusion surgery.

## Supplementary Material

Supplementary data
