# Supplementary material for: A self-organising biomimetic collagen/nano-hydroxyapatite-glycosaminoglycan scaffold for spinal fusion
Source: J Mater Sci. Author manuscript; Available in PMC 2018 Jul 3. (PMC6029624; doi:10.1007/s10853-017-1229-9)
Supplement: Supplementary data [file NIHMS974423-supplement-Supplementary_data.pdf]

Supplementary Material 1

**Primer Name**	**Sequence**
ALP f	ACCACCACGAGAGTGAACCA
ALP r	CGTTGTCTGAGTACCAGTCCC
BGLAP (Osteocalcin) f	TCACACTCCTCGCCCTATT
BGLAP (Osteocalcin) r	TGAAAGCCGATGTGGTCAG
BMP-2 f	GGGTGGAATGACTGGATTGT
BMP-2 r	GTCAAGGTACAGCATCGAGATAG
GAPDH f	ACAGTCAGCCGCATCTTCTT
GAPDH r	AATGAAGGGGTCATTGATGG
RUNX2 f	CACTATCCAGCCACCTTTACTT
RUNX2 r	AGCGTCAACACCATCATTCT
SP7 (Osterix) f	CCTCTGCGGGACTCAACAAC
SP7 (Osterix) r	AGCCCATTAGTGCTTGTAAAGG
TGFβ 1 f	GTGGAAACCCACAACGAAATC
TGFβ 1 r	GTGTCCAGGCTCCAAATGTA
TBP f	GAATATAATCCCAAGCGGTTTG
TBP r	ACTTCACATCACAGCTCCCC
β ACTIN f	GTTTGAGACCTTCAACACCC
β ACTIN r	ATGTCACGCACGATTTCCC
UBC f	ATTTGGGTCGCGGTTCTTG
UBC r	TGCCTTGACATTCTCGATGGT
